# Circular noncoding RNA circMBOAT2 is a novel tumor marker and regulates proliferation/migration by sponging miR-519d-3p in colorectal cancer

**DOI:** 10.1038/s41419-020-02869-0

**Published:** 2020-08-14

**Authors:** Xiaolong Tang, Guorui Sun, Qingsi He, Chao Wang, Jingbo Shi, Lei Gao, Jianhong Ye, Yahang Liang, Hui Qu

**Affiliations:** 1 Department of General Surgery, Qilu Hospital of Shandong University, Jinan, 250012 China; 2 Qilu Medical College of Shandong University, Jinan, 250011 China

**Keywords:** Non-coding RNAs, Colorectal cancer

## Abstract

Colorectal cancer (CRC) is a common malignant tumor with a poor prognosis. However, its pathogenesis has not been fully elucidated, accounting for poor overall survival. Circular RNA (circRNA) is a class of noncoding RNAs discovered many years ago. Only recently have they been re-evaluated for their important roles in the regulation of gene expression. Studies have confirmed that circRNAs have important biological functions in a variety of malignant tumors. This study aimed to characterize one circRNA derived from the *MBOAT2* gene and termed it circMBOAT2, which has been reported to promote prostate cancer progression. CircMBOAT2 is highly expressed in both CRC tissues and serum samples, and has a correlation with tumor stage. The receiver-operating characteristic curves suggested that circMBOAT2 acted as a novel diagnostic tumor marker in CRC. Univariate and multivariate analyses showed that the levels of circMBOAT2 in tissues were independent prognostic markers of CRC. Further functional studies revealed that circMBOAT2 served as a microRNA (miRNA) sponge of miR-519d-3p and promoted the proliferation, migration, and invasion of CRC cells. Also, circMBOAT2 regulated cell proliferation and migration by competitively binding to miR-519d-3p and targeting troponin-associated protein (TROAP) in CRC cells. These results suggested that circMBOAT2 might be a novel potential biomarker of CRC.

## Introduction

Colorectal cancer (CRC) is one of the most common malignant tumors worldwide, ranking fourth in terms of both cancer incidence and mortality worldwide^1^. The molecular mechanism of the development and progression of CRC remains unclear, accounting for poor survival. Circular RNA (circRNA) is a noncoding RNA with a closed-loop structure^2^. With the progress of RNA deep sequencing technology and bioinformatics in recent years, it has been gradually revealed that circRNA is endogenous, abundant, conserved, and stable in mammalian cells, and has many molecular biological functions^3^. Moreover, circRNAs are abundant in eukaryotic cells, and their abundance in eukaryotic cells exceeds that of other competing endogenous RNAs (ceRNAs)^4^. They are largely composed of exons, mostly exist in the cytoplasm, and have highly conserved and tissue-specific structures. Most of the circRNAs are noncoding RNAs, but a small number of intron-derived RNA molecules have coding functions^5^. Many studies have found a close association of circRNAs with malignant tumors and their important roles in the pathogenesis of tumors^6–9^.

Some circRNAs have been reported to be abnormally expressed in CRC. However, the detailed molecular mechanism of circRNAs in CRC remains largely unknown^10,11^. A cohort of seven cancerous tissues (including CRC) from Gene Expression Omnibus (GEO) datasets (GSE77661) was used to identify potential candidates for the single patient classifier genes during the exploratory phase of study^12^. Bioinformatics analysis and quantitative reverse transcription-polymerase chain reaction (qRT-PCR) were used in CRC tissues to focus on a circRNA termed as circMBOAT2, which has been reported to promote prostate cancer progression^13^. The expression of circMBOAT2 was upregulated in CRC tissues, and the expression level was significantly associated with tumor stage and prognosis, implying the involvement of circMBOAT2 in CRC. This study aimed to investigate the clinical significance and molecular mechanism of circMBOAT2 in CRC, thus providing a new target for the diagnosis and treatment of CRC.

## Results

### CircMBOAT2 was aberrantly upregulated in CRC tissues

**Fig. 1:**
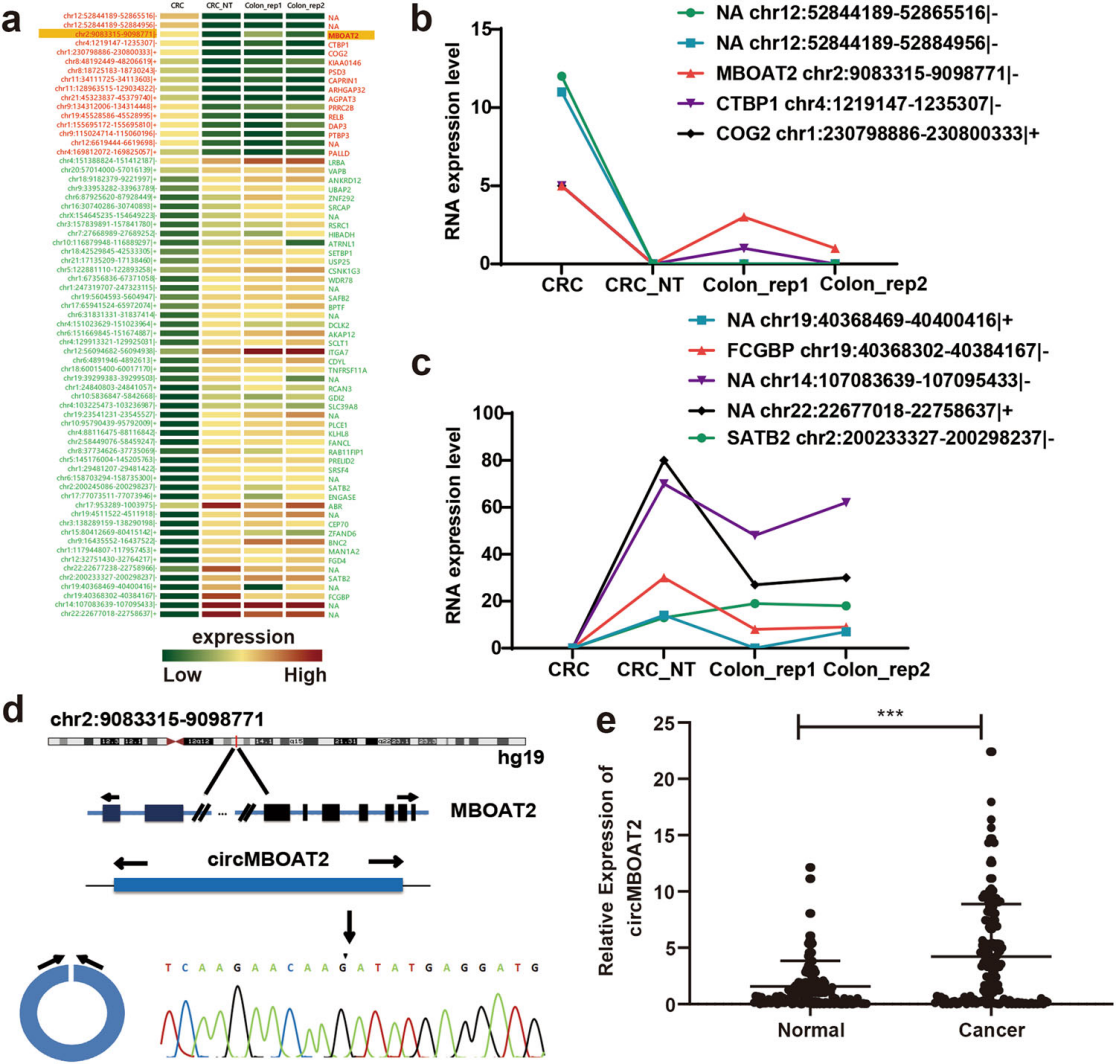
CircMBOAT2 was upregulated in CRC tissues. **a** Differential expression of circRNA in CRC tissues and matched normal tissues (CRC: tumor tissues, *n* = 1; CRC_NT: adjacent normal tissues, *n* = 1; CRC_rep: normal mucosa tissues, *n* = 2). **b** Five most upregulated circRNAs in CRC tissues analyzed by RNA deep sequencing technology and bioinformatics. **c** Five most downregulated circRNAs in CRC tissues analyzed by RNA deep sequencing technology and bioinformatics. **d** Genomic loci of the *MBOAT2* gene and circMBOAT2. The expression of circMBOAT2 was detected by qRT-PCR and validated by Sanger sequencing. **e** Expression of circMBOAT2 in colorectal cancer tissues and adjacent normal tissues (*n* = 169, the 2^−ΔΔCt^ method).

The transcriptome sequencing data of CRC tissues from GEO datasets (GSE77661, Fig. 1a) were reanalyzed. To identify the differentially expressed circRNAs, the GSE77661 dataset contained a clump of CRC circRNA sequencing data, including data of tumor tissues (*n* = 1), adjacent normal tissue (*n* = 1), and normal mucosa tissues (*n* = 2) were analyzed. The identification and quantification of human circRNAs were performed as previously described^12^. Five most upregulated circRNAs (Fig. 1b) and five most downregulated circRNAs (Fig. 1c) in CRC tissues were selected for further experiments. Also, a qRT-PCR assay of these 10 circRNA molecules was performed in CRC tumors and adjacent normal tissues (data not shown). Then, the study focused on a circRNA located at chr2:9083315–9098771, which was stably upregulated in CRC tumor tissues. Its ID was hsa_circ_0007334 in the CircBase database (http://www.circbase.org/) with a transcriptional source gene of *MBOAT2*, which was termed as circMBOAT2 (Fig. 1d). The expression of circMBOAT2 in 169 paired CRC tissues (including tumor tissues and adjacent normal tissues) was examined using qRT-PCR to detect the difference in the expression of circMBOAT2 in tumor tissues. The results showed that the expression of circMBOAT2 was significantly upregulated in CRC tissues compared with matched normal tissues (*P* < 0.01, Fig. 1e).

### CircMBOAT2 was an independent prognostic marker of CRC

**Fig. 2:**
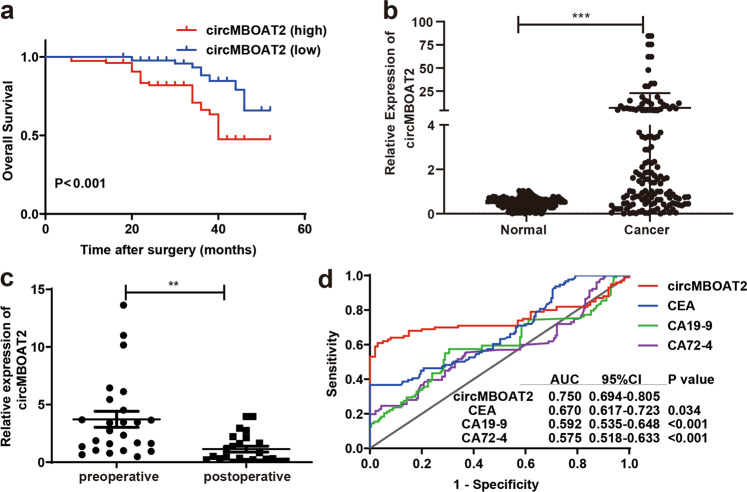
CircMBOAT2 was a diagnostic tumor marker of CRC. **a** Kaplan–Meier analysis of the correlation between the expression of circMBOAT2 and overall survival in 169 patients with CRC patients (log-rank test). **b** Expression of circMBOAT2 in the serum of patients with CRC (*n* = 107) and healthy people (*n* = 100, 2^−ΔΔCt^ method). **c** Expression of circMBOAT2 in preoperative and postoperative sera of patients with CRC (*n* = 40). **d** *P* values show the area under the ROC of the circMBOAT2 signature versus CEA/CA19-9/CA72–4 in the serum of patients with CRC. **P < 0.01, ***P < 0.005, ****P < 0.001.

The overall survival (OS) curve was plotted by the Kaplan–Meier method based on the expression levels of circMBOAT2 in CRC tumor tissues to investigate whether circMBOAT2 could be used as a prognostic marker. As shown in Fig. 2a, patients with CRC having high expression levels of circMBOAT2 had lower 5-year OS than those with low levels of circMBOAT2 (median survival 20 months vs. 46 months; *P* < 0.001, log-rank test). As shown in Table 1, the expression of circMBOAT2 was significantly associated with distant metastasis (*P* = 0.011), pTNM stage (*P* < 0.001), and lymphovascular invasion (*P* = 0.002). As shown in Table 2, pT stage [hazard ratio (HR) = 2.45, 95% confidence interval (CI): 1.14–5.3; *P* = 0.023], distant metastasis (HR = 5.95, 95% CI: 2.58–13.73; *P* < 0.001), pTNM stage (HR = 3.29, 95% CI: 1.27–8.53; *P* = 0.014), and expression levels of circMBOAT2 (HR = 2.27, 95% CI: 1.00–5.12; *P* = 0.029) were independent prognostic factors for OS of patients with CRC. Moreover, patients with a high expression level of circMBOAT2 had a worse prognosis compared with those with a low expression level in serum. However, the differences were not statistically significant (*P* = 0.166, Supplementary Fig. 1).

**Table 1:** Correlation between the expression of circMBOAT2 and clinical pathological factors in CRC tissues.

Variables	No. of patients	circMBOAT2 (high)	circMBOAT2 (low)	*χ*^2^	*P*
*Gender*				1.33	0.250
Male	116	57 (73.08)	59 (64.84)		
Female	53	21 (26.92)	32 (35.16)		
*Age (year)*				1.43	0.232
≤60	95	40 (51.28)	55 (60.44)		
>60	74	38 (48.72)	36 (39.56)		
*Tumor location*				0	0.981
Colon	67	31 (39.74)	36 (39.56)		
Rectum	102	47 (60.26)	55 (60.44)		
*Tumor diameter (cm)*				1.45	0.228
≤5	57	30 (38.46)	27 (29.67)		
>5	112	48 (61.54)	64 (70.33)		
*Tumor differentiation*				1.62	0.203
Poor	34	19 (24.36)	15 (16.48)		
Well/moderate	135	59 (75.64)	76 (83.52)		
*pT stage*				0.01	0.936
T1–3	123	57 (73.08)	66 (72.53)		
T4	46	21 (26.92)	25 (27.47)		
*pN stage*				0.36	0.551
N0	93	41 (52.56)	52 (57.14)		
N1–2	76	37 (47.44)	39 (42.86)		
*Distant metastasis (M stage)*				6.45	**0.011**
M0	155	67 (85.9)	88 (96.7)		
M1	14	11 (14.1)	3 (3.3)		
*pTNM stage*				13.27	**<0.001**
I–II	73	22 (28.21)	51 (56.04)		
III–IV	96	56 (71.79)	40 (43.96)		
*Perineural invasion*				0.01	0.910
Yes	19	9 (11.54)	10 (10.99)		
No	150	69 (88.46)	81 (89.01)		
*Lymphovascular invasion*				9.8	**0.002**
Yes	8	8 (10.26)	0 (0)		
No	161	70 (89.74)	91 (100)		
*Postoperative recurrence or metastasis*				0.01	0.906
Yes	34	16 (20.51)	18 (19.78)		
No	135	62 (79.49)	73 (80.22)		

All *P* values < 0.05 were marked in bold print.

**Table 2:** Univariate and multivariate overall survival analyses of clinicopathological factors in patients with CRC.

Variables	Univariate analysis		Multivariate analysis	
	HR (95% CI)	*P* value	HR (95% CI)	*P* value
Gender (male vs. female)	0.44 (0.17–1.13)	0.088		
Age (≤60 years vs. >60 years)	0.85 (0.43–1.68)	0.647		
Tumor diameter (≤5 cm vs. >5 cm)	1.96 (0.99–3.85)	0.052		
Tumor location (colon vs. rectum)	1.50 (0.76–2.96)	0.240		
Differentiation (well/moderate vs. poor)	0.89 (0.39–2.06)	0.794		
pT stage (T1–3 vs. T4)	2.02 (1.02–4.01)	**0.043**	2.45 (1.14–5.3)	**0.023**
pN stage (N0 vs. N1–2)	1.60 (0.80–3.20)	0.188		
Distant metastasis (M0 vs. M1)	7.11 (3.37–15.00)	**<0.001**	5.95 (2.58–13.73)	**<0.001**
pTNM stage (I–II vs. III–IV)	5.28 (2.15–13.01)	**<0.001**	3.29 (1.27–8.53)	**0.014**
circMBOAT2 expression (low vs. high)	3.50 (1.63–7.55)	**0.001**	2.27 (1.00–5.12)	**0.029**

All *P* values < 0.05 were marked in bold print.

### CircMBOAT2 could be a diagnostic tumor marker of CRC

This study further investigated the expression of circMBOAT2 in the serum of patients with CRC and healthy people. Also, qRT-PCR was used to detect the expression level of circMBOAT2 in the serum of 107 patients with CRC and 100 healthy people. The results showed that the expression of circMBOAT2 was significantly upregulated in the serum of patients with CRC compared with healthy people (*P* < 0.001, Fig. 2b). The expression of circMBOAT2 was significantly higher in preoperative serum than in postoperative serum (*P* < 0.01, Fig. 2c). The receiver-operating characteristic (ROC) curves were used to investigate whether circMBOAT2 could be used as a diagnostic tumor marker. The area under the ROC curve (AUC) showed a significant difference in the expression levels of circMBOAT2 compared with those of carcinoembryonic antigen (CEA), carbohydrate antigen 19-9 (CA19-9), and carbohydrate antigen 72-4 (CA72-4) (AUC = 0.750 vs. 0.670/0.592/0.575, all *P* < 0.05, Fig. 2d). The results showed that the expression of circMBOAT2 in serum had a better diagnostic value compared with that of CEA/CA19-9/CA72-4 alone. However, a combination of three or four markers did not improve the AUC compared with circMBOAT2 alone (0.666 vs. 0.750; 0.746 vs. 0.750, Supplementary Fig. 2).

### CircMBOAT2 could promote the proliferation of CRC cells in vitro and in vivo

**Fig. 3:**
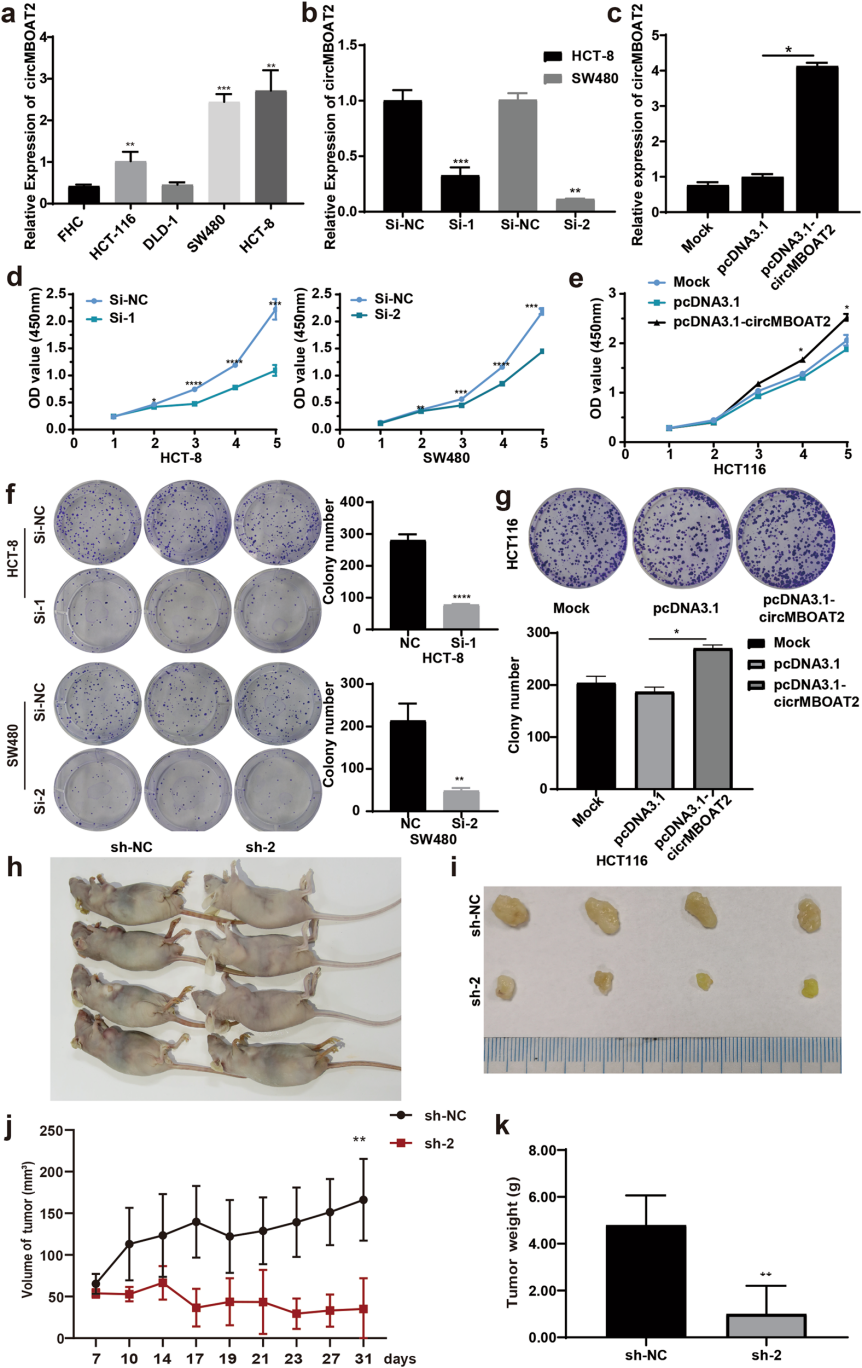
CircMBOAT2 promoted the proliferation of CRC cells in vitro and in vivo. **a** Expression of circMBOAT2 in human colorectal normal epithelial cells (FHC) and four colorectal cancer cell lines (HCT-116, DLD-1, SW480, and HCT-8). **b** After transfection with si-circMBOAT2 RNA, the expression level of circMBOAT2 significantly reduced. **c** Expression levels of circMBOAT2 were detected in HCT-116 cells transfected with circMBOAT2 overexpression vector and empty vector (mock) by qRT-PCR. **d** CCK-8 assay showed that circMBOAT2 knockdown could inhibit the growth of HCT-8 and SW480 cells. **e** CCK-8 assay showed that the overexpression of circMBOAT2 could promote the growth of HCT-116 cells. **f** Colony formation assay showed that circMBOAT2 knockdown could inhibit the proliferation of HCT-8 and SW480 cells. **g** Colony formation assay showed that the overexpression of circMBOAT2 could promote the proliferation of HCT-116 cells. **h** Xenograft tumors were established by injecting SW480 cells stably expressing sh-circMBOAT2 compared with sh-NC (*n* = 4, each group). Representative images of nude mice are shown. **i** The sh-circMBOAT2 stably expressing group and the sh-NC group were used for xenograft tumorigenesis assay. The tumors of nude mice were harvested and evaluated after 4 weeks. **j**, **k** After subcutaneous injection of SW480 cells, tumor growth curves with the stable silencing of circMBOAT2 or the negative control are shown. The volumes (**j**) and tumor weights (**k**) were measured every 3 days after inoculation. **P < 0.01, ***P < 0.005, ****P < 0.001.

qRT-PCR was used to detect the expression level of circMBOAT2 in human colorectal epithelial cells (FHC) and colorectal cancer cell lines HCT-8, DLD-1, SW480, and HCT-116. The results showed that the expression levels of circMBOAT2 significantly increased in HCT-8, SW480, and HCT-116 (*P* < 0.05) (Fig. 3a). CRC cell lines HCT-8 and SW480 were transfected with circMBOAT2 siRNAs (Fig. 3b) and the cell line HCT-116 was transfected with the overexpression vector of pcDNA3.1-circMBOAT2 (Fig. 3c) to assess the biological function of circMBOAT2. The results of cell counting kit 8 (CCK-8) assay showed that the growth trend of HCT-8 and SW480 cells was lower in the si-circMBOAT2 group than in the control group after 3 days of cell culture (Fig. 3d), and the growth of HCT-116 cells followed an opposite trend after transfection with the overexpression vector (Fig. 3e). The results of colony formation experiments indicated that the interference with the expression of circMBOAT2 could inhibit the proliferation of CRC cell lines HCT-8 and SW480 (Fig. 3f), while the overexpression of circMBOAT2 could promote the proliferation of HCT-116 cells (Fig. 3g). These results indicated that circMBOAT2 could promote the proliferation of CRC cells in vitro.

Xenograft tumor models in nude mice were further established using SW480 cells transfected with sh-negative control (sh-NC) or sh-circMBOAT2 to determine whether circMBOAT2 regulated cell proliferation in vivo. The stable knockdown of circMBOAT2 was performed in SW480 cells by infection with lentivirus-carrying shRNAs (Supplementary Fig. 3). The cells infected with sh-NC or sh-circMBOAT2 were injected subcutaneously into male BALB/c nude mice, and xenograft tumors were harvested 4 weeks after injection (Fig. 3h, i). The results showed that the volumes and weights of xenograft tumors with circMBOAT2 knockdown were significantly lower than those in the control group (tumor volume, 155.2 ± 35.1 mm^3^ vs. 48.2 ± 11.1 mm^3^, *P* = 0.004, Fig. 3j; tumor weight, 4.76 ± 1.25 g vs. 1.43 ± 0.31 g, *P* = 0.007, Fig. 3k).

### CircMBOAT2 could promote the invasion and migration of CRC cells in vitro

**Fig. 4:**
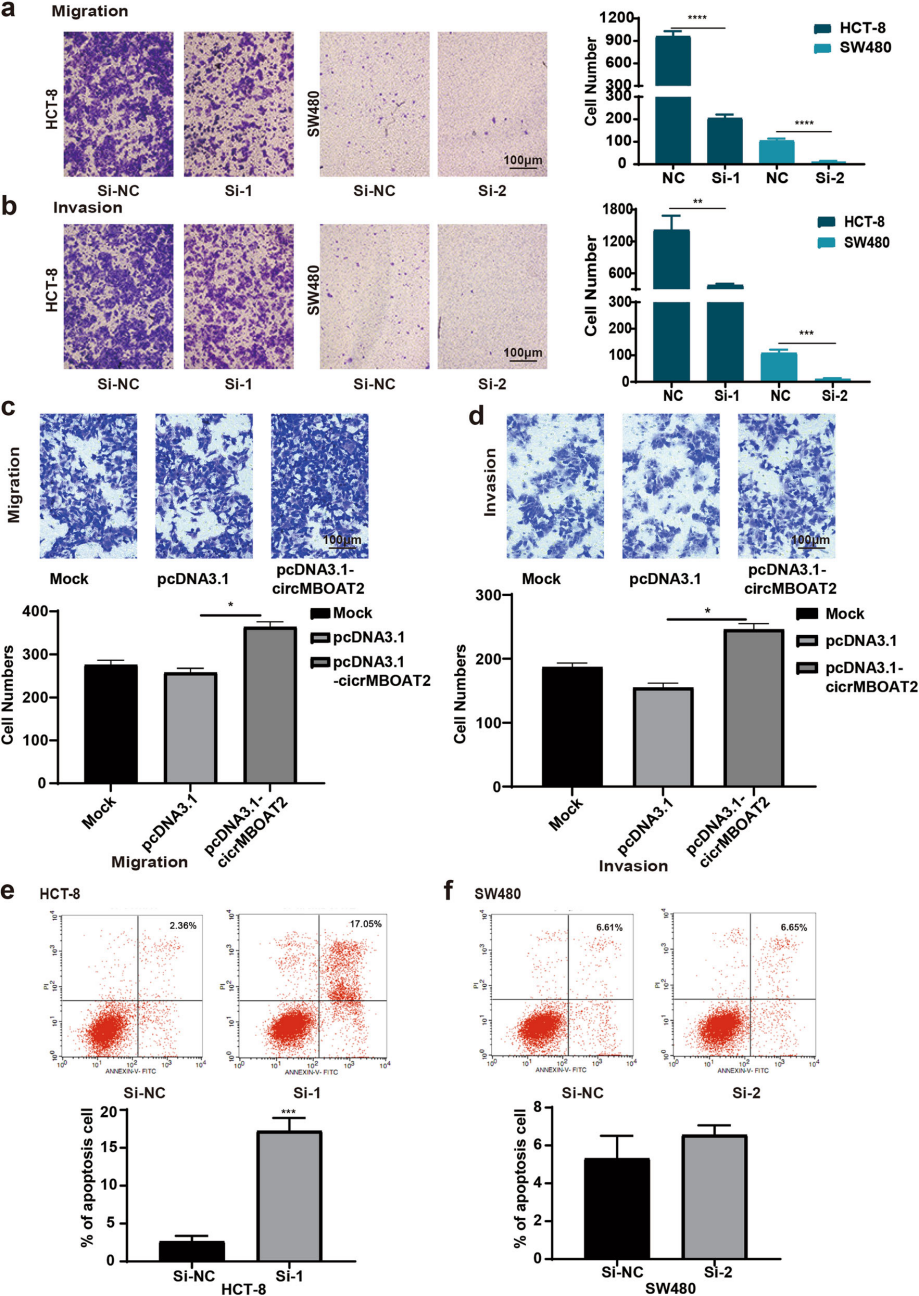
Knockdown of circMBOAT2 could inhibit the invasion and migration of CRC cells in vitro. **a** After circMBOAT2 knockdown, the invasive capability of HCT-8 and SW480 cells decreased significantly. **b** Interference with the expression of circMBOAT2 significantly inhibited the migratory capability of HCT-8 and SW480 cells. **c**, **d** Cell migratory (**c**) and invasive (**d**) capabilities were promoted in HCT-116 cells after transfection with pcDNA3.1-circMBOAT2. **e**, **f** Flow cytometry results showed that circMBOAT2 knockdown promoted apoptosis in HCT-8 cells (**c**) but not in SW480 cells (**d**). **P < 0.01, ***P < 0.005, ****P < 0.001.

The control and circMBOAT2 siRNAs were transfected into CRC cell lines HCT-8 and SW480, respectively. The results showed that the migratory and invasive capabilities of HCT-8 and SW480 cells significantly decreased after the inhibition of circMBOAT2 (Fig. 4a, b). After transfection with the overexpression vector pcDNA3.1-circMBOAT2, the migratory and invasive capabilities of HCT-116 cells significantly increased (Fig. 4c, d). The results suggested that circMBOAT2 promoted the invasion and migration of CRC cells. Flow cytometry was used to detect the apoptotic capability of HCT-8/SW480 cells after interference with circMBOAT2. The number of apoptotic cells significantly increased after HCT-8 cells interfered with circMBOAT2 (17.05%), indicating that the interference with the expression of circMBOAT2 could promote the apoptosis of HCT-8 cells (Fig. 4e). However, the interference with circMBOAT2 (6.65%) had no influence on SW480 cells (Fig. 4f). CCK-8 assay and migration assay were conducted in HCT-8 and SW480 cells to prove that these biological functions of circMBOAT2 were independent of the source gene *MBOAT2*. The results showed that MBOAT2 could not promote cell proliferation or migration in vitro (Supplementary Fig. 4).

### CircMBOAT2 could competitively bind to downstream miRNAs

**Fig. 5:**
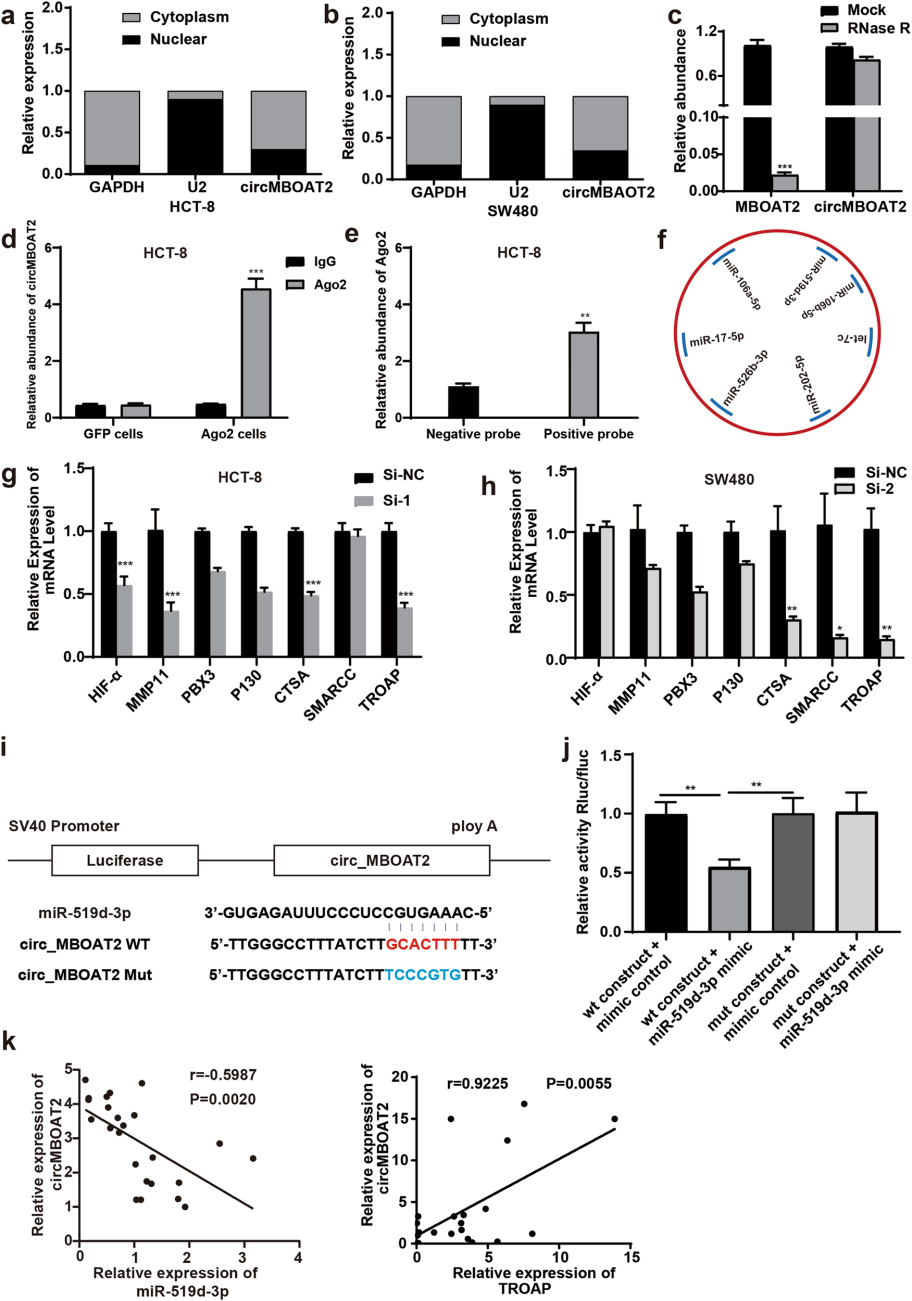
CircMBOAT2 could serve as miRNA sponge. **a**, **b** qRT-PCR analysis of nuclear and cytoplasmic RNAs in HCT-8 (**a**) and SW480 (**b**) cells. **c** qRT-PCR analysis of circMBOAT2 and MBOAT2 RNA after treatment with RNase R enzyme in HCT-8 cells. **d** RNA immunoprecipitation (RIP) assay for the amount of circMBOAT2 in HCT-8 cells stably expressing GFP or Ago2. **e** RNA pull-down assay showed relative abundance of Ago2 after treated with biotinylated circMBOAT2 positive and negative probe in HCT-8 cells. **f** A schematic diagram showing the seven miRNAs which are most likely binding to circMBOAT2. **g**, **h** After the knockdown of circMBOAT2, the change in the expression levels of the target mRNAs were examined by qRT-PCR in HCT-8 (**g**) and SW480 (**h**) cells. **i** Schematic illustration shows the putative binding sites of miR-519d-3p with respect to circMBOAT2. **j** After co-transfection with circMBOAT2-WT or circMBOAT2-MUT and mimics, inhibitor, or NC, the relative luciferase activities were detected in 293T cells. **k** Expression of circMBOAT2 and miR-519d-3p in CRC tumor tissues had a negative correlation as detected by qRT-PCR. **l** Expression of circMBOAT2 and TROAP in CRC tumor tissues had a positive correlation as detected by qRT-PCR. **P < 0.01, ***P < 0.005, ****P < 0.001.

The qRT-PCR analysis of nuclear and cytoplasmic RNA revealed that circMBOAT2 was mainly localized in the cytoplasm of HCT-8 (Fig. 5a) and SW480 cells (Fig. 5b). After the extracted total RNA was treated with RNase R enzyme, the linear gene *MBOAT2* was degraded, while *circMBOAT2* was not (Fig. 5c). RNA immunoprecipitation (RIP) for Argonaute 2 (Ago2) was performed in HCT-8 cells stably expressing Ago2 or green fluorescent protein (GFP), revealing that endogenous circMBOAT2 pulled down from Ago2 cells was specifically enriched by qRT-PCR (Fig. 5d). On the other side, RNA pull-down assay showed relative abundance of Ago2 treated with biotinylated circMBOAT2-positive probe was significantly upregulated, implying that Ago2 could combine to circMBOAT2 (Fig. 5e). These results suggested that circMBOAT2 could bind to Ago2 and act as miRNA sponge. Furthermore, the TargetScan 6.0 and miRanda software were used to predict the downstream miRNAs that competitively bound to circMBOAT2, so as to explore the molecular mechanisms by which circMBOAT2 regulated the function of CRC cells (Supplementary Table 1). According to the information given by the software and references searched on the PubMed database (https://pubmed.ncbi.nlm.nih.gov/), seven target miRNAs of circMBOAT2 were selected for further analysis (Fig. 5f). Then, the change in the expression level of the target mRNA was detected using qPCR. The results showed that the transfection of si-circMBOAT2 affected the expression of multiple downstream target genes, and TROAP was the mRNA with the most significantly decreased expression downstream of the target gene of miR-519d-3p^14^ in both HCT-8 (*P* < 0.005, Fig. 5g) and SW480 cells (*P* < 0.01, Fig. 5h). Bioinformatics analysis suggested that circMBOAT2 and miR-519d-3p shared potential binding sites on miR-519d-3p (Fig. 5i). Then, miR-519d-3p was used for luciferase assays. The full-length circMBOAT2-wild type (WT) and miR-519d-3p mimics were co-transfected into 293T cells with a luciferase reporter gene. The miR-519d-3p mimics significantly reduced luciferase reporter gene activity compared with control miRNA mimics (Fig. 5j). Moreover, the Pearson correlation analysis showed that the expression of circMBOAT2 had a negative correlation with miR-519d-3p (Fig. 5k) and a positive correlation with TROAP (Fig. 5l) in CRC tumor tissues, as detected by qRT-PCR. These results suggested that circMBOAT2 could competitively bind to miR-519d-3p.

### CircMBOAT2 promoted the proliferation and migration of CRC cells via the miR-519d-3p/TROAP axis

**Fig. 6:**
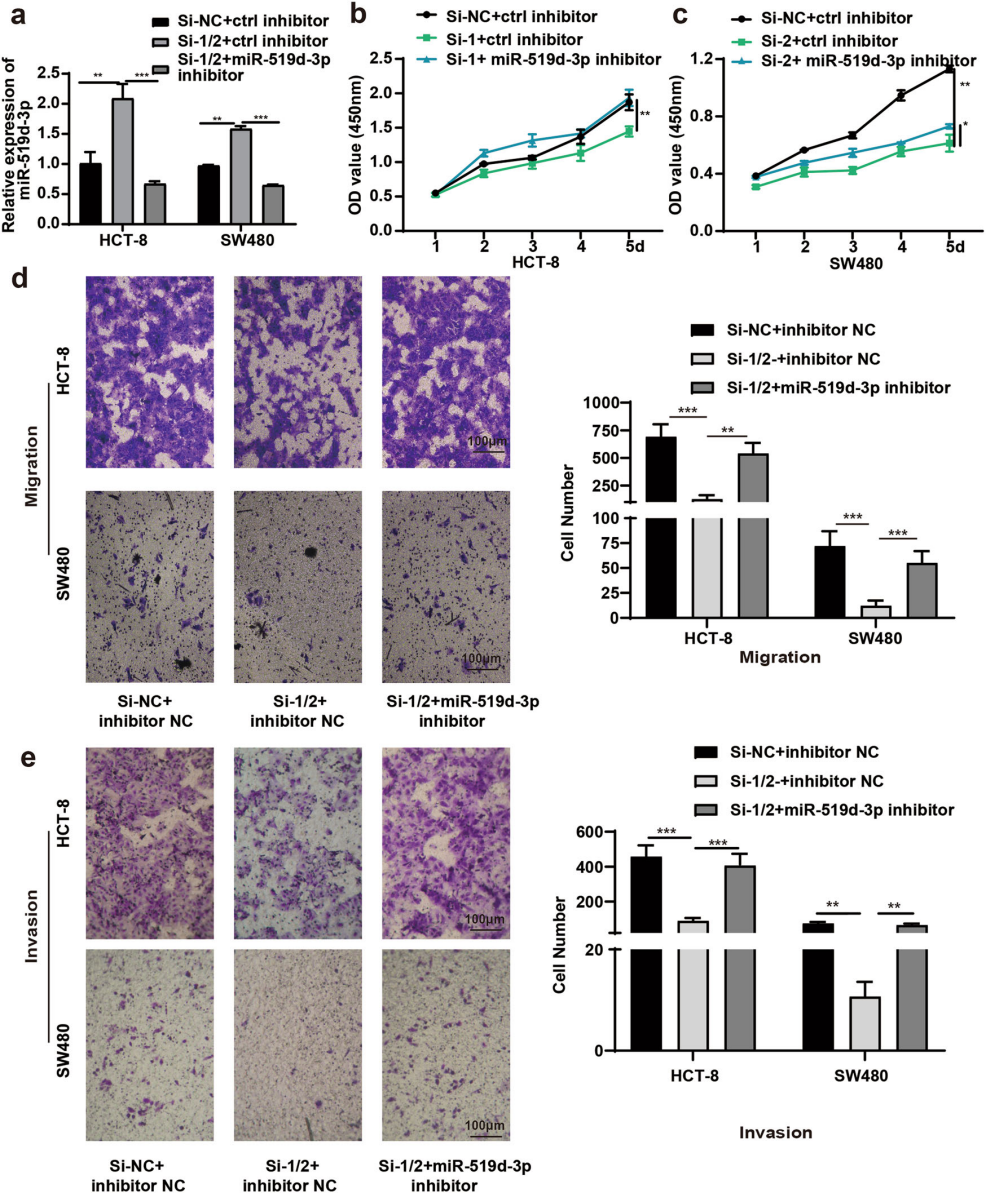
CircMBOAT2 regulated CRC cells via miR-519d-3p. **a** Relative expression of miR-519d-3p after interfering with circMBOAT2 and adding miR-519d-3p inhibitor. **b**, **c** After knockdown of circMBOAT2 and inhibition of miR-519d-3p, the proliferation capability of HCT-8 (**b**) and SW480 (**c**) cells was restored. **d**, **e** After knockdown of circMBOAT2 and inhibition of miR-519d-3p, the migration (**d**) and invasion (**e**) capabilities of HCT-8 and SW480 cells were restored. **P < 0.01, ***P < 0.005, ****P < 0.001.

**Fig. 7:**
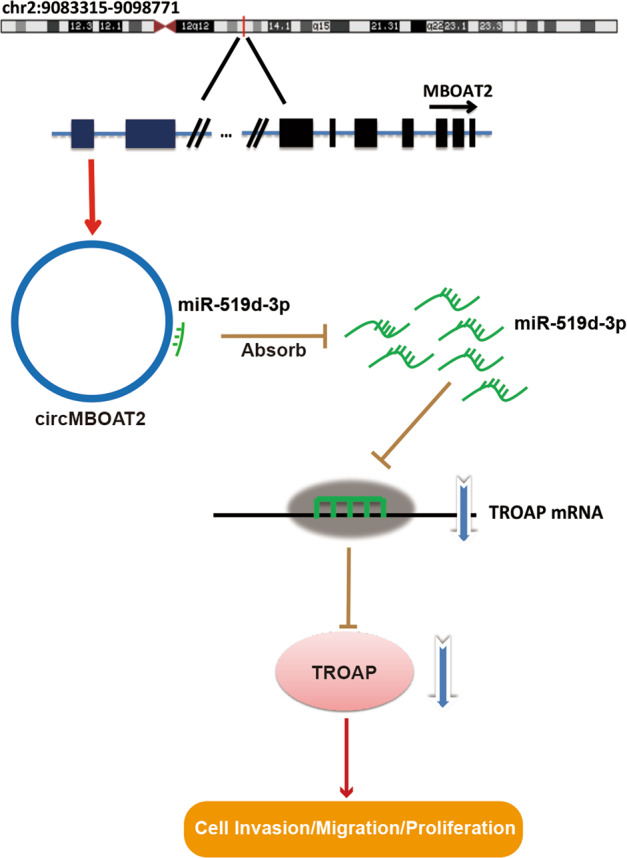
Schematic diagram of the molecular mechanism of circMBOAT2. CircMBOAT2 promoted the proliferation and migration in CRC cells via miR-519d-3p/TROAP axis.

Based on the results showing a tumor-promoting effect of circMBOAT2 on CRC cells, the study next evaluated whether the effects of circMBOAT2 were mediated by miR-519d-3p. Therefore, the cells were co-transfected with circMBOAT2 siRNA and miR-519d-3p inhibitor (Fig. 6a). After inhibiting the expression of miR-519d-3p, the proliferative (Fig. 6b, c), migratory (Fig. 6d), and invasive (Fig. 6e) capabilities of HCT-8 and SW480 cells caused by the decreased expression of circMBOAT2 were restored. The miR-519d-3p was reported to be a tumor suppressor by targeting TROAP in CRC cells^14^. Furthermore, the knockdown of circMBOAT2 promoted the expression of miR-519d-3p (Fig. 6a) and inhibited the expression of TROAP in CRC cells (Fig. 5f). It was inferred that circMBOAT2 promoted the proliferation and migration of CRC cells via the miR-519d-3p/TROAP axis (Fig. 7).

## Discussion

Despite the improvement in diagnostic and therapeutic treatments in recent years, many patients are still in an advanced stage at the time of diagnosis and miss the best time for treatment due to the lack of effective molecular markers for the early diagnosis of CRC^15^. Therefore, studying the molecular mechanism of CRC development and exploring new early diagnostic molecular markers is necessary to improve the prognosis of patients with CRC. The discovery of circRNA enriches the understanding of noncoding RNA families, and its unique circular structure endows it with special molecular biological functions, providing a new perspective in the diagnosis and treatment of CRC^16^.

CircRNAs are abnormally expressed in various tumor tissues, and their expression level is related to clinicopathological factors. For example, hsa_circ_0001946 was upregulated in lung adenocarcinoma^8^. Hsa_circ_0001649 was downregulated in hepatocellular carcinoma and significantly correlated with tumor stage^17^. The present study confirmed that the expression of circMBOAT2 was upregulated in CRC tissues and significantly correlated with the tumor stage of patients with CRC. Furthermore, circMBOAT2 was found to be correlated with prognosis and was an independent prognostic biomarker in patients with CRC. CircRNAs have a covalently closed-loop structure, which makes circRNAs more stable than other linear RNA molecules^18^. The expression of circMBOAT2 was evaluated in the serum samples of patients with CRC undergoing radical resection and healthy people. The results indicated that the expression of circMBOAT2 was stably upregulated in the serum of patients with CRC and reduced after the surgery. ROC curves showed that circMBOAT2 was a better diagnostic biomarker than CEA/CA19–9/CA72-4, implying that circMBOAT2 was stably expressed in the blood and had the potential to be a diagnostic tumor marker of CRC. Moreover, recent study showed that highly expressed circMBOAT2 may associate with cardiac diseases^19^.

The functional studies of circMBOAT2 showed that the knockdown of circMBOAT2 in HCT-8 and SW480 cells could inhibit the proliferation, migration, and invasion of CRC cells, while the overexpression of circMBOAT2 in HCT-116 cells exerted opposite effects. These results suggested that circMBOAT2 could promote cell proliferation, migration, and invasion in CRC. These biological functions are consistent with those of circMBOAT2 in prostate cancer^13^. Moreover, experiments showed that the biological functions of circMBOAT2 were independent of the source gene *MBOAT2*, as MBOAT2 did not show any of these functions (Supplementary materials). Self-replication, apoptosis resistance, tumor invasion, and distant metastasis are the main features of malignant tumors. The currently used chemotherapeutic drugs act on tumor cells to achieve a tumor-suppressing effect^20^. In addition, studying the role of circMBOAT2 provides a better understanding of the etiology and development of CRC, hence guiding the early diagnosis of CRC and improving the prognosis of patients with CRC. The special molecular biology functions of circRNAs have also prompted research on CRC. Zhu et al.^21^ showed that circular BANP modulated cell proliferation in CRC. Guo et al.^22^ found that Hsa_circ_0000069 was upregulated in the CRC cell line and promoted tumor cell proliferation, invasion, and metastasis.

Recently, circRNAs were reported to act as miRNA sponges; they could control the expression of miRNA and downstream protein functions^23^. The number of these binding regions in several known circRNAs far exceeds the ones in well-known ceRNAs. Therefore, they can exert more powerful adsorption functions, specifically bind to miRNAs and affect the downstream target genes of miRNAs, and play regulatory roles in malignant tumors^24^. This effect is completely analogous to that of the ceRNA molecule, indicating that it has an RNA sponge adsorption function^25^. Recent studies on ceRNAs have been carried out in various malignant tumors such as lung cancer^26^, gastric cancer^9^, and liver cancer^27^. CircHIPK3 molecules in a variety of tumor tissues competitively bind to miR-124, miR-152, miR-193a, miR-29a, and other miRNAs^28^. CircPVT1 acts as a sponge of downstream miR-125 molecules and promotes the proliferation of gastric cancer cell lines^29^. Hsa_circ_0067934 was upregulated in esophageal squamous cell carcinoma and promoted cell proliferation^30^.

As mentioned earlier, circRNAs can act as RNA sponges, absorbing the corresponding miRNAs and affecting downstream target gene activity^31^. Therefore, it is speculated that circRNA molecules may also play a regulatory role in tumor cells, affecting tumor proliferation and invasion. Thus, it is important and meaningful to study the mechanism of action of circRNAs in tumors. RNAs regulate target gene expression by binding to Argonaute 2 (Ago2), which is an essential component of RNA-induced silencing complex^32^. In the present study, the RIP assay and RNA pull-down was performed in HCT-8 cells, confirming that circMBOAT2 could bound to Ago2 and acts as miRNAs sponge. Using dual-luciferase reporter assay, the present study revealed that circMBOAT2 could sponge miR-519d-3p in CRC cells. MicroRNA (miRNA) has been shown to serve as a vector by combining with the seed region and sequence in the 3′-untranslated region (UTR) of the target mRNA, and hence regulate gene expression after transcription^33^. MiRNAs are involved in a wide array of biological processes, including cell growth, apoptosis, differentiation, and hematopoiesis. MiR-519d-3p has been reported as a tumor suppressor in CRC^14^, pancreatic cancer^34^, cervical cancer^35^, and breast cancer^36^. It inhibits cell proliferation and migration by targeting TROAP in CRC^14^. Troponin-associated protein (TROAP, Tastin) is a proline-rich protein with 778 amino acids; it is needed for bipolar spindle assembly and centrosome integrity during mitosis^37^. Troponin has been reported to promote CRC cell invasion through a mechanism involving HMGB1/RAGE^38^. Moreover, downregulated TROAP in liver cancer may play a critical role through p21 and p27^39^. The upregulation of TROAP in CRC tissues has been confirmed; it promotes the proliferation and migration of CRC cells^14^. These findings suggested that the dysregulation of TROAP expression might play a key role in the development of CRC. In the present study, a series of experiments, such as the Pearson correlation analysis and the rescue experiments, were conducted to prove the correlations among circMBOAT2, miR-519d-3p, and TROAP. Finally, the study proved that circMBOAT2 could promote proliferation and migration via sponging miR-519d-3p and finally regulate the expression TROAP in CRC.

Based on the previous results, it was concluded that circMBOAT2 had a sponge mechanism in CRC cells; it absorbed downstream miRNAs and further regulate the transcription of the corresponding target gene *TROAP*. Hence, circMBOAT2 promoted the proliferation, migration, and invasion of CRC cells in vitro and in vivo. This study investigated the molecular mechanism of interaction between circMBOAT2 and downstream miRNAs and target genes. It increased the possibility of circMBOAT2 serving as a tumor marker for the diagnosis of CRC and its role in the development of CRC. This study might lay the experimental foundation for further research and provide new ideas and basis for the diagnosis and treatment of CRC.

## Materials and methods

### Human tissue specimens and serum samples

All CRC tissue specimens and serum samples were obtained from the Department of Gastrointestinal Surgery, Qilu Hospital of Shandong University, between April 2013 and December 2015. Tumor tissues and matched adjacent normal tissues were collected at the same time after the surgery. Fresh venous blood samples from patients with CRC and healthy individuals matched according to age and sex were collected. Patients who received neoadjuvant chemotherapy/radiotherapy were excluded. The informed consent was obtained from all patients. This study was approved by the ethics committee of Qilu Hospital of Shandong University.

### RNA extraction and quantitative real-time PCR

Total RNA was extracted using a TRIzol LS Reagent (Invitrogen, MA, USA) kit. The purity and quantity of total RNA were then determined using DeNovix DS-11 (DeNovix, DE, USA). The cDNA was synthesized from 1 μg total RNA using a Prime-Script RT kit (Takara, Tokyo, Japan). Real-time PCR was conducted with an SYBR Premix Ex Taq System (Takara, Tokyo, Japan), using CFX96 (Bio-Rad, CA, USA). All primers were designed and synthesized by CloudSeq Co., Ltd. (Shanghai, China) (Table 3). Glyceraldehyde 3-phosphate dehydrogenase (GAPDH) was used as an internal reference. The data were analyzed using the ΔCt method. All results were expressed as the mean ± standard deviation (SD) of three independent experiments.

**Table 3:** Primers, siRNA, shRNA, and probes sequences used in this article.

Primers used for qRT-PCR	
GAPDH F	GGCCTCCAAGGAGTAAGACC
GAPDH R	AGGGGAGATTCAGTGTGGTG
circMBOAT2 F	GGAGTGGAGAACATGCACAA
circMBOAT2 R	AAGGCAAAGAGTTGGCACAC
miR-519d-3p F	TGCGGCAAAGTGCCTCCCTTT
miR-519d-3p R	CCAGTGCAGGGTCCGAGGTA
HIF-α F	ACGTTCCTTCGATCAGTTGTCACC
HIF-α R	GGCAGTGGTAGTGGTGGCATTAG
MMP11 F	GGAGAAGACGGACCTCACCTACAG
MMP11 R	CAGTACCTGGCGAAGTCGATCATG
PBX3 F	CAAAGAAACATGCCCTGAACTG
PBX3 R	GCTGAGACCTGTTTTCTCTTTG
P130 F	GAAACTTCACTGTTTCTGCTGT
P130 R	AGCAGGTGATAAGAATTGACC
CTSA F	GGGCAATGGACTCTCCTCCT
CTSA R	GACCAAAGCCTGTTCCCCAG
SMARCC F	TAATTACCAAGTTGACCCGGAA
SMARCC R	GTAAATGTCAGTACGGAGACCA
TROAP F	GAGAAATGTCACATACCAGGGA
TROAP R	GTTTTAGGTTGAACGGTGAGAC
U2 F	ATCTGAAACGCGACTCACCG
U2 R	GACGGAGCAAGCCCCTATTC
MBOAT2 F	AGTGCAAGATAAAGGCCCAAA
MBOAT2 R	TGATCATCATAGGAGTGGAGAA
*siRNA oligonucleotides*	
si-circMBOAT2–1#	5′-GAGAACAUGCACAAGUCAA-3′
	3′-CUCUUGUACGUGUUCAGUU-5′
si-circMBOAT2–2#	5′-CAUGCACAAGUCAACUUUG-3′
	3′-GUACGUGUUCAGUUGAAAC-5′
si-negative control	5′-GCGACGAUCUGCCUAAGAUdTdT-3′
	3′-AUCUUAGGCAGAUCGUCGCdTdT-5′
*shRNA oligonucleotides*	
sh-circMBOAT2	5′-CATGCACAAGTCAACTTTG-3′
	3′-CAAAGTTGACTTGTGCATG-5′
sh-negative control	5′-TTCTCCGAACGTGTCACGTAA-3′
	3’-TTACGTGACACGTTCGGAGAA-5’
Biotin-circMBOAT2 positive probe	5′-biotin-AAACAAAGAGTTGGCACACTACAAAGTTG ACTTGTGCATGTTCTCCACTCC-3′
Biotin-circMBOAT2 negative probe	5′-biotin-AAAGGAGTGGAGAACATGCACAAGTCAAC TTTGTAGTGTGCCAACTCTTTG-3′

### Cell culture and transfection

HCT-8 and DLD-1 cells were cultured in RPMI-1640 medium plus 10% (v/v) fetal bovine serum (FBS). HCT-116 and SW480 cells were cultured in DMEM medium plus 10% (v/v) FBS. The medium and FBS were purchased from Gibco (NY, USA). The cell lines were incubated in a humidified atmosphere with 5% CO_2_ at 37 °C. The siRNAs, miR-519d-3p mimics, control mimics, and miR-519d-3p inhibitor used in this study were designed and synthesized by Suzhou Ribo Life Science Co., Ltd. (Suzhou, China). The siRNA sequences used in the study are shown in Table 3. The overexpression vectors pcDNA3.1, pcDNA3.1-circMBOAT2, and pcDNA3.1-MBOAT2 were constructed by Hanbio Life Science Co., Ltd. (Shanghai, China). The cells were transfected using Lipofectamine 2000 (Invitrogen, MA, USA). The cells were harvested after 48 h for subsequent experiments.

### Colony formation assay and CCK-8 assay

For the colony formation assays, the cells were counted after trypsinization following transfection for 48 h, and 500 cells were added to each well in a 6-well plate. After cultured for 8–14 days, the cells were counted using 0.05% crystal violet staining. Then, the cell colonies were counted and analyzed. Cell proliferation was assessed using a CCK-8 assay. After 48 h of cell transfection, the cells were seeded in 96-well plates (2–3 × 10^3^ cells/well), and the plates were cultured in an incubator. After cell attachment, 10 μL of CCK-8 solution (Apexbio, TX, USA) was added to each well on days 1–5. After incubation for 2 h, the absorbance was measured at 450 nm using an enzyme-linked immunosorbent assay. All results were expressed as the mean ± SD of three independent experiments.

### Transwell assay

After transfection for 48 h, HCT-8 and SW480 cells were suspended in serum-free RPMI-1640 or DMEM. For migration assay, 200 μL of suspension containing 1 × 10^5^ cells was added to the Transwell chamber (Corning, NY, USA). For invasion assay, 200 μL of suspension containing 1 × 10^5^ cells was added to the Transwell chamber covered with Matrigel (BD Biosciences, NJ, USA). RPMI-1640 or DMEM containing 20% serum was added to the lower chamber. After 24 s, nonmigratory or noninvasive cells were removed. The cells migrating through the membrane were counted under a microscope (Olympus, Tokyo, Japan) after fixing and staining.

### Cell apoptosis assay

Cell apoptosis assay was performed using an Annexin V–FITC/PI kit (Absin, Shanghai, China). After 72 h of cell transfection, the cells floating in the supernatant were collected, and the adherent cells were resuspended by trypsin digestion. Each group comprising 1 × 10^6^ cells was stained, and apoptosis was detected by flow cytometry (Beckman, FL, USA).

### Isolation of nuclear and cytoplasmic fractions

HCT-8 was resuspended on ice in 0.3% NP-40/NIB-250 buffer (15 mM TrisCl, pH 7.5, 60 mM KCl, 15 mM NaCl, 5 mM MgCl_2_, 1 mM CaCl_2_, and 250 mM sucrose) to isolate nuclear and cytoplasmic fractions. The fractions were treated with a protease inhibitor for 10 min. After centrifugation at 600*g* for 5 min at 4 °C, the resulting supernatant was collected as a cytoplasmic fraction and mixed with an equal volume of TRIsure reagent (Bioline, London, UK). The pellet was then washed with NIB-250, and the nuclei were lysed in the TRIsure reagent.

### RNA-binding protein immunoprecipitation and RNA pull-down assay

The experiment was carried out following the flow instructions of an RNA-binding protein immunoprecipitation (RIP) kit (Merck Millipore, MA, USA). RIP assay was performed following the manufacturer’s protocols. The final sample was dissolved in 10–20 μL of diethylpyrocarbonate (DEPC) water, and the samples were tested by qRT-PCR. RNA pull-down assay was carried out according to the flow of a Pierce magnetic RNA-protein pull-down kit (Thermo Fisher, MA, USA). The biotinylated circMBOAT2 positive and negative probe were synthesized by CloudSeq Co., Ltd. (Shanghai, China) (sequences are shown in Table 3). The RNA pull-down assay was performed following the manufacturer’s protocols. Finally, the protein was eluted with 50 μL of elution buffer and identified by protein profiling.

### Bioinformatics analysis

CummeRbund package (http://compbio.mit.edu/cummeRbund/) was used to identify and filter circRNAs from the GEO datasets GSE77661. The target miRNAs were searched using TargetScan 6.0 and miRanda. According to the context score given by TargetScan, and structure score & free energy given by miRanda, the target miRNAs of circMBOAT2 were selected (Supplementary Table 1). The PubMed databases (https://pubmed.ncbi.nlm.nih.gov/) were used to search and identify seven miRNAs which have been reported to be tumor suppressors in CRC^14,40–45^. The change in the expression of target miRNA and mRNA was examined by qRT-PCR.

### Dual-luciferase reporter assay

The plasmids pMIR-circMBOAT2-WT and pMIR-circMBOAT2-MUT were constructed by Hanbio Life Science Co., Ltd. (Shanghai, China). The circMBOAT2 linear sequence was cloned into the pMIR-circMBOAT2-WT 3′-UTR region, and the circMBOAT2-mut linear sequence was cloned into the pMIR-circMBOAT2-MUT 3′-UTR region. The plasmid and miR-519d-3p mimics were co-transfected into 293T cells to measure fluorescence intensity. After 48 h of incubation, the luciferase activities were quantified with a dual-luciferase reporter assay (Promega, WI, USA).

### Xenograft and orthotopic models of CRC in mice

The sequence of siRNA was inserted into a lentiviral vector (pHBLV-U6-MCS-CMV-ZsGreen-PGK-PURO) to stably knockdown circMBOAT2 (named sh-circMBOAT2). sh-circMBOAT2 and an empty lentiviral vector (sh-negative control, sh-NC) were constructed by Hanbio Life Science Co., Ltd. (Shanghai, China). The sh-circMBOAT2 and sh-NC sequences are shown in Table 3. Lentiviral vectors were constructed containing the *GFP* gene and a puromycin resistance gene. SW480 cells were transfected and selected with puromycin (1.2 μg/mL; Sigma-Aldrich, MO, USA). The animal experiments were approved by the ethics committee of Qilu Hospital of Shandong University. Male BALB/c mice of 4 weeks age were purchased from Vital River Laboratory Animal Technology Co., Ltd. (Beijing, China). Mice were randomly divided into two groups. SW480 cells (1 × 10^6^ cells) transfected with sh-NC (group #1) or sh-circMBOAT2 (group #2) were injected into the right side of the backs of the nude mice. After 9 days, the longest and shortest diameters of tumors were measured every 2 days with calipers, which were, respectively, recorded as L and W, and the tumor volumes were calculated as *V* = *L* × *W*^2^ × 0.5.

### Statistical analysis

The expression levels of circMBOAT2 in tissues and serum correlated with various clinicopathological factors. A follow-up was conducted telephonically or through posts, and the patients were followed up for postoperative recurrence, distant metastasis, and OS. Data were analyzed using the *t* test and one-way analysis of variance or the nonparametric Kruskal–Wallis test. The Pearson correlation coefficient was used to analyze the correlations. The Kaplan–Meier method and log-rank test were used to calculate the OS curves. The Cox proportional-hazards regression model was used in the univariate and multivariate analyses. ROC curves were generated. Statistical analysis was performed using SPSS 20.0 software (IBM, IL, USA). A *P* value less than 0.05 was considered statistically significant.

## Supplementary information

Supplementary materials

Supplementary Figure 1

Supplementary Figure 2

Supplementary Figure 3

Supplementary Figure 4

Supplementary Table 1
