# Supplementary material for: Circular noncoding RNA circMBOAT2 is a novel tumor marker and regulates proliferation/migration by sponging miR-519d-3p in colorectal cancer
Source: Cell Death Dis. 2020 Aug 14;11(8):625. doi: 10.1038/s41419-020-02869-0 (PMC7429508; doi:10.1038/s41419-020-02869-0)
Supplement: Supplementary file 1 — Supplementary materials [file 41419_2020_2869_MOESM1_ESM.docx]

**Supplementary materials**

**Supplementary Figure 1.** Kaplan–Meier analysis of the correlation between circMBOAT2 expression in serum and overall survival (*n* = 95, log-rank test).

**Supplementary Figure 2.** AUC of different CRC tumor markers (single or combined).

**Supplementary Figure 3.** Stable knockdown of circMBOAT2 was performed in SW480 cells by the infection with a lentivirus carrying shRNAs.

**Supplementary Figure 4.** MBOAT2 could not promote cell proliferation or migration *in vitro.* (a) Expression level of MBOAT2 in CRC normal (*n* = 41) and tumor (*n* = 45) tissues detected by qRT-PCR. (b) Expression level of MBOAT2 in TCGA datasets (normal sample, *n* = 44; cancer sample, *n* = 568). (c) MBOAT2 mRNA expression levels were detected in HCT-8 and SW480 cells transfected with MBOAT2 overexpression vector and empty vector (mock) by qRT-PCR. (d) CCK-8 assay showed that MBOAT2 overexpression could not promote the growth of HCT-8 or SW480 cells. (e) Migratory capabilities were not promoted in HCT-8 or SW480 cells after transfection with pcDNA3.1-MBOAT2.

**Supplementary Table 1.** Prediction of binding miRNAs from the TargetScan and miRanda for circMBOAT2.
