# Supplementary figures and images for: Circular noncoding RNA circMBOAT2 is a novel tumor marker and regulates proliferation/migration by sponging miR-519d-3p in colorectal cancer
Source: Cell Death Dis. 2020 Aug 14;11(8):625. doi: 10.1038/s41419-020-02869-0 (PMC7429508; doi:10.1038/s41419-020-02869-0)

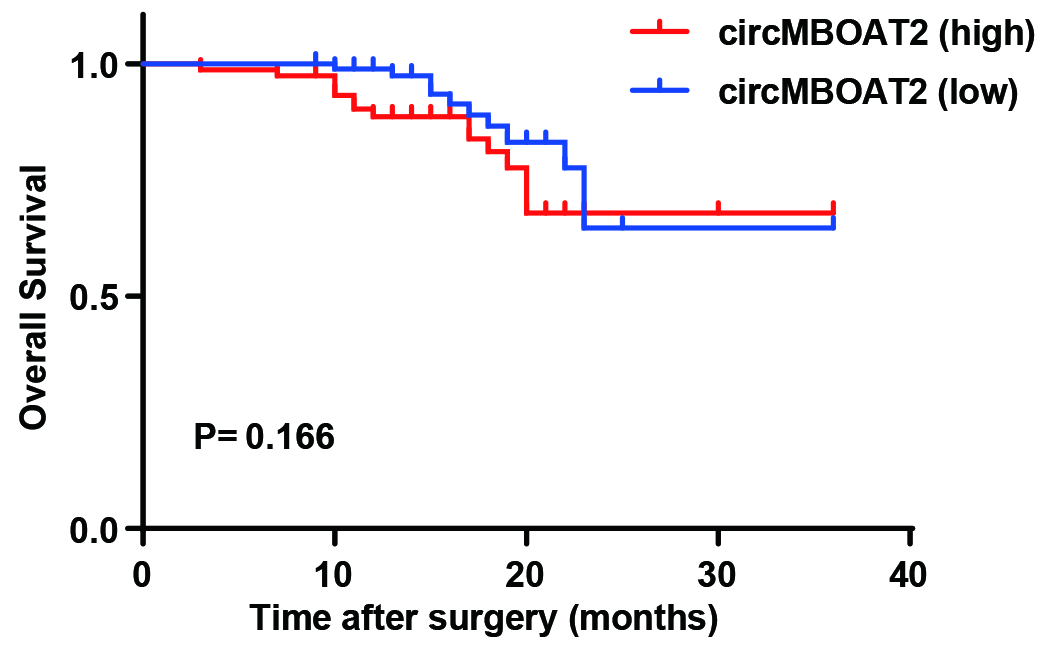

Supplement: Supplementary file 2 — Supplementary Figure 1 [file 41419_2020_2869_MOESM2_ESM.tif]

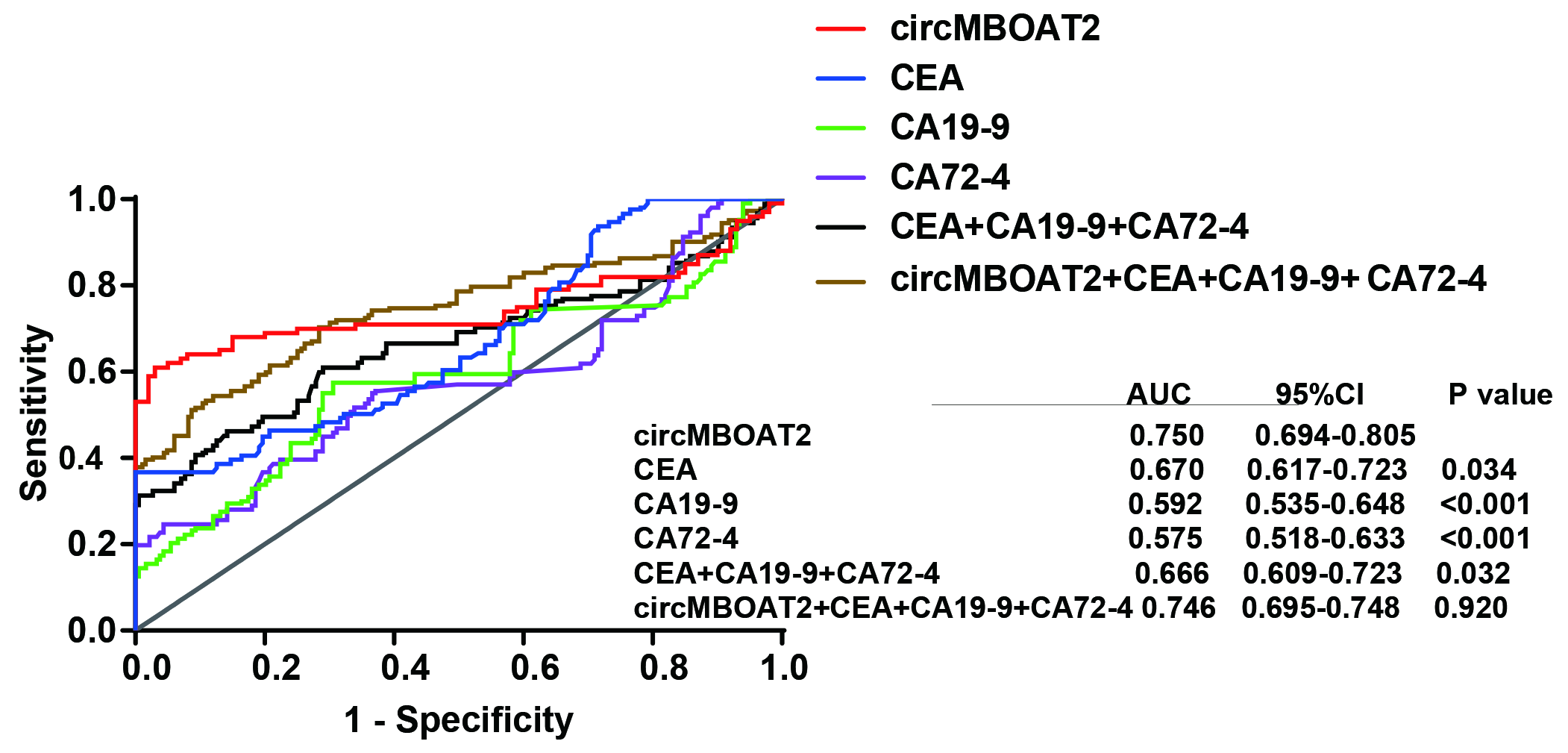

Supplement: Supplementary file 3 — Supplementary Figure 2 [file 41419_2020_2869_MOESM3_ESM.tif]

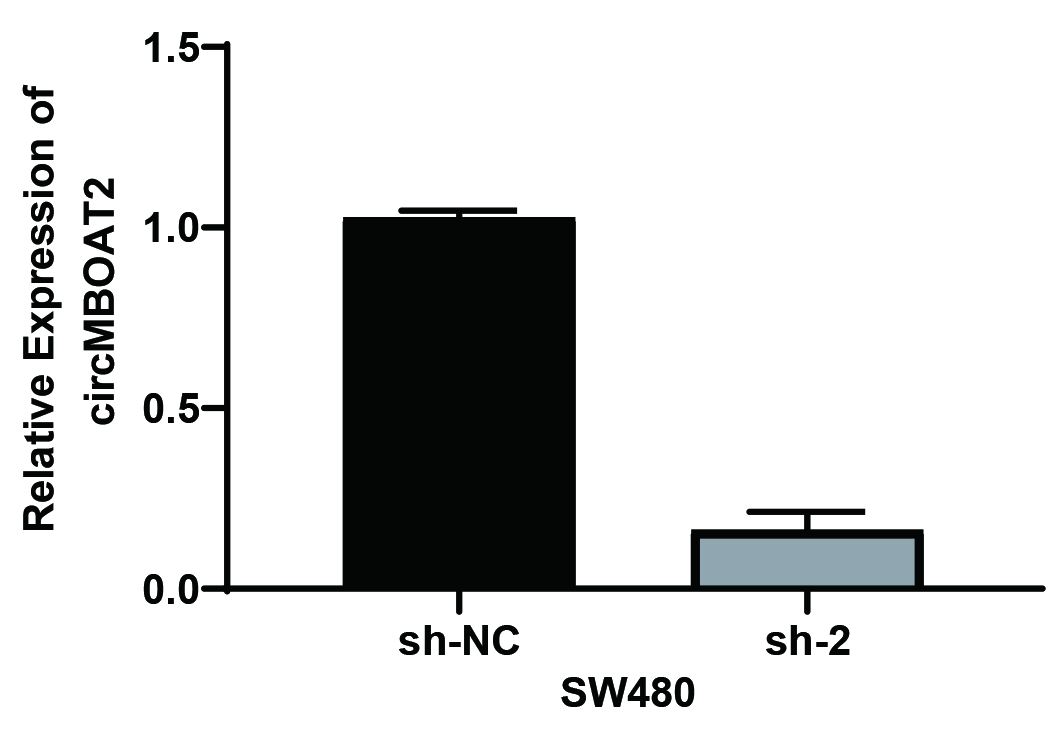

Supplement: Supplementary file 4 — Supplementary Figure 3 [file 41419_2020_2869_MOESM4_ESM.tif]

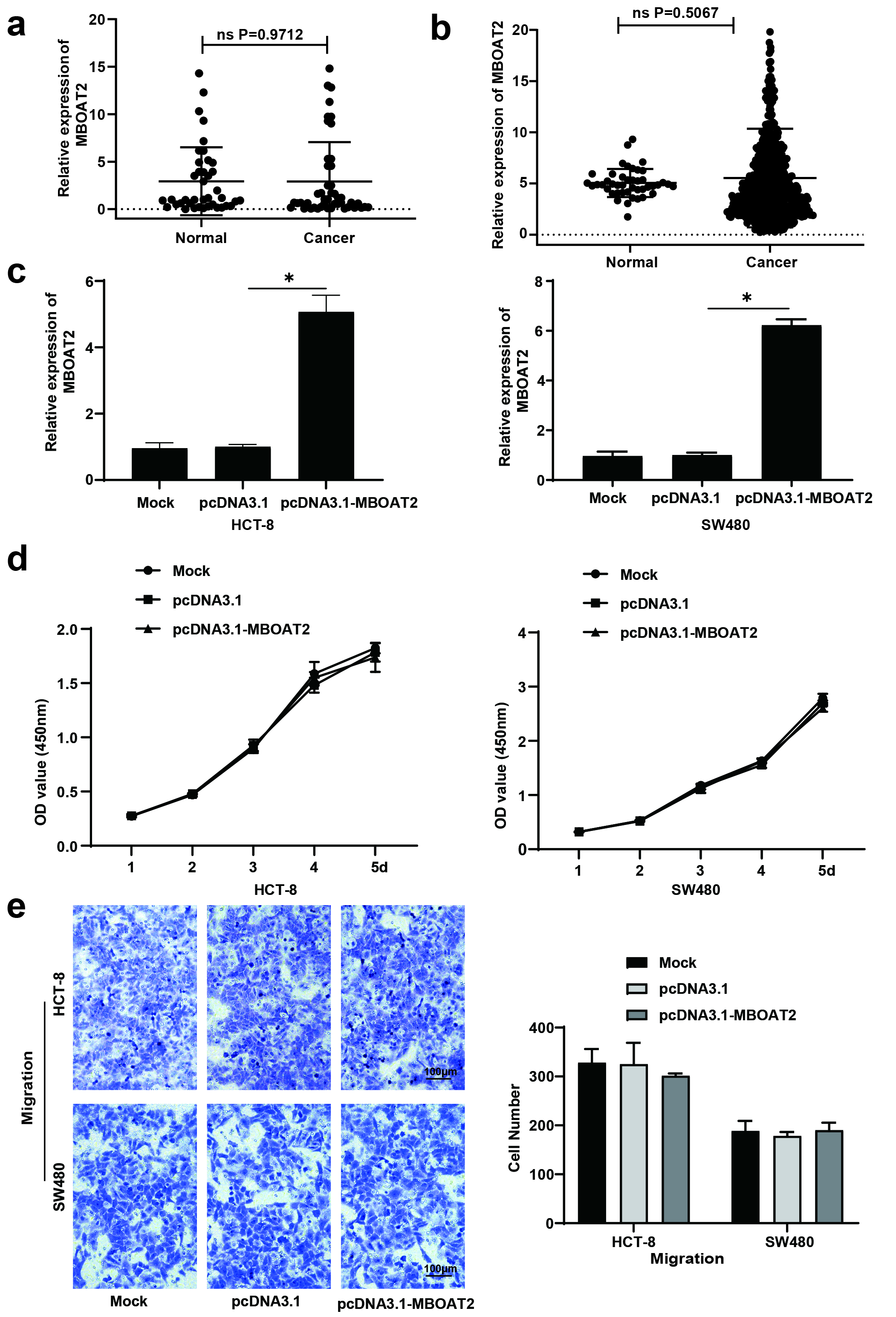

Supplement: Supplementary file 5 — Supplementary Figure 4 [file 41419_2020_2869_MOESM5_ESM.tif]
